# Altered expression of ADM and ADM2 by hypoxia regulates migration of trophoblast and HLA-G expression^†^

**DOI:** 10.1093/biolre/ioaa178

**Published:** 2020-09-30

**Authors:** Changdai Gu, Sohae Park, Jin Seok, Hee Yeon Jang, Yong Ju Bang, G I Jin Kim

**Affiliations:** Metabolic and Biomolecular Engineering National Research Laboratory, Systems Metabolic Engineering and Systems Healthcare (SMESH) Laboratory, Department of Chemical and Biomolecular Engineering (BK21 Plus Program), Institute for the BioCentury, Korea Advanced Institute of Science and Technology (KAIST), Daejeon, Republic of Korea; Department of Biomedical Science, CHA University, Gyeonggi-do, Republic of Korea; Department of Biomedical Science, CHA University, Gyeonggi-do, Republic of Korea; Department of Biomedical Science, CHA University, Gyeonggi-do, Republic of Korea; Seoulin Bioscience Co., Ltd, Seongnam-si, Gyeonggi-do, Republic of Korea; Department of Biomedical Science, CHA University, Gyeonggi-do, Republic of Korea

**Keywords:** adrenomedullin, adrenomedullin2, trophoblast, preeclampsia, hypoxia, invasion, placenta, human leukocyte antigen-G

## Abstract

Preeclampsia (PE) is a placental disorder caused by endothelial dysfunction via trophoblast inadequate invasion activity. Adrenomedullin (ADM) and ADM2 are multifunctional peptides that can support vascular activity and placental growth. However, correlation between ADMs and trophoblast functions is currently unclear. The objective of this study was to analyze changes in expression of ADMs in placenta and HTR-8/SVneo trophoblast cells under hypoxia and their effects on invasion activity of trophoblast cells and expression of HLA-G. In placental tissues of PE, expression levels of ADM and HLA-G were significantly increased (*P* < 0.05) whereas expression of ADM2 was decreased compared to that in normal term placenta. Under hypoxia, expression levels of ADM, ADM2, and HLA-G and invasion ability of trophoblast cells were increased in hypoxia-inducible factor-1 (HIF-1α)- dependent manner (*P* < 0.05). Treatment with ADMs agonists reduced HIF-1α activity whereas enhanced invasion ability under hypoxia. However, they were not changed after cotreatment of ADMs and HIF-1α inhibitor, YC-1, although expression levels of invasion-related genes MMP2, MMP9, and Rac1 were altered (*P* < 0.05). ADMs also increased HLA-G expression under normoxia whereasADM2 or cotreatment of ADMs under hypoxia attenuated HLA-G expression (*P* < 0.05). Our findings demonstrate that altered expression of ADMs plays a critical role in placental physiology, especially in trophoblast invasion and immune-modulation under hypoxia.

## Teaser

Summary sentence Altered expression of ADMs plays a critical role in placental physiology, especially in trophoblast invasion and immune-modulation under hypoxia.

## Introduction

Trophoblast cells derived from trophectoderm of blastocysts play an important role in embryo implantation and placental development [1, 2]. Trophoblast cells can invade into the maternal endometrium and remodel arterial blood vessels in the uterine wall. This is known as spiral arteries transformation. Trophoblast causes angiogenesis and vasodilation through adrenomedullin (ADM) and vascular endothelial growth factor (VEGF). It induces the immune tolerance by expression of human leukocyte antigen G (HLA-G) and secretion of hormones [2–5]. Therefore, trophoblast cells can regulate early implantation and placental development for interactions between fetus and mother during pregnancy. In early trophoblast differentiation, low oxygen tension is one of physiological factors for organogenesis and a key regulator of cellular events, eventually provoking oxidative stress in trophoblast [6]. Especially, hypoxia has significant effects on placental development mediated by hypoxia-inducible factor 1-alpha (HIF-1α) which activates gene transcription in response to varying oxygen concentrations [7]. Caniggia et al. have reported that HIF-1α is elevated during early pregnancy and it appears to be crucial for mediating biological effects of oxygen on early trophoblast differentiation [8]. Persistent oxidative stress in trophoblast can lead to inadequate invasion of trophoblast, results in infertility and obstetric diseases such as preeclampsia (PE) and intrauterine growth restriction (IUGR) [9, 10].

PE is a critical obstetric disease characterized by endothelial dysfunction, eventually increasing the risk of fetal and maternal mortality and morbidity [11–13]. PE is majorly associated with abnormal trophoblast differentiation which causes poor invasion ability of trophoblast, resulting in impaired spiral artery remodeling, inadequate uteroplacental circulation, and maternal hypertension [14]. Trophoblast invasion in PE is regulated by various secreting factors under hypoxic conditions during pregnancy. Placental hypoxia is a central pathogenic factor of PE due to vascular remodeling by abnormal trophoblast invasion. However, the precise molecular mechanisms as well as biomarkers for diagnosis are not well elucidated yet. Thus, studies on molecular mechanism and mining of biomarkers are should be needed.

ADM, a multifunctional peptide hormone, is widely expressed in many kinds of tissues. It supports physiologic regulation of vasculature and placental growth [3, 15, 16]. ADM is a 52-amino acid peptide with structural homology to calcitonin gene-related peptide (CGRP). It is important in normal placental function [16]. Recently, ADM2, also known as intermedin (IMD), is newly discovered [PMID: 17965749, 18]. It is closely related to ADM that belongs to the CGRP family sharing a single seven-transmembrane G protein-coupled receptor, calcitonin receptor-like receptor (CRLR) and receptor activity-modifying proteins (RAMPs) [17–19]. Like ADM, ADM2 is expressed in many organs of the body, especially trophoblast cells. It plays an important role in the regulation of trophoblast invasion, migration, and HLA class I, G (HLA-G) expression in human placenta [20, 21]. Previous studies have also shown that ADM plays a significant role in the proliferation, migration, and angiogenesis of tumor cells [19, 22]. In addition to vascular activities of ADM and ADM2 in trophoblast cells, migration-related proteins, matrix metalloproteinases (MMPs), and Rho family appear to play a critical role in mediating trophoblast invasion [17, 23]. It has been reported that ADM is regulated by HIF-1-dependent pathway under hypoxic conditions [19, 24]. However, the regulation mechanism of ADM and ADM2 in trophoblast invasion under hypoxic conditions involving HLA-G and expression of migration-related genes has not been reported yet. Therefore, the objective of the present study was to investigate the significance of ADM, ADM2, and HLA-G expression patterns in PE placenta and how their expression pattern might change in an extravillous trophoblast cell line (i.e., HTR-8/SVneo) under hypoxic condition. Furthermore, the correlation between expression of ADM, ADM2, HIF-1α, MMP2, MMP9, Rho family, and HLA-G and trophoblast invasion activity according to hypoxia was evaluated.

## Materials and methods

### Placenta and blood collection in human PE

The collection of human PE placental tissues and blood was approved by the Institutional Review Board of CHA General Hospital, Seoul, Korea. All participants provided written, informed consent prior to sample collection (IRB No.: 2006-12). Mean age of 15 normal pregnant (NPE) women was 34 ± 2 years. Their pregnancy length was 38 ± 2.5 weeks. Their systolic and diastolic blood pressures were 115 ± 3 mm Hg and 72 ± 2 mm Hg, respectively. Albuminuria was not detected in NPE women. Fifteen pregnant women in the 3rd trimester PE were 30.2 ± 1.1 years old. Their pregnancy length was 37 ± 3.4 weeks. Their systolic and diastolic blood pressures were 134 ± 7 mm Hg and 91 ± 2 mm Hg, respectively. An average of proteins in albuminuria through dipstick detection were over two positive.

### Cell culture

HTR-8/SVneo, a trophoblast cell line, was provided by Dr Graham (Queen’s University, Kingston, Ontario, Canada). HTR-8/SVneo cells were cultivated in Roswell Park Memorial Institute 1640 medium (RPMI-1640; Hyclone, GE healthcare life sciences, Seoul, Korea) supplemented with 1% penicillin–streptomycin (Pen-Strep; Gibco, Rockville, MD, USA) and 5% Fetal Bovine Serum (FBS; Gibco, Rockville, MD, USA) at 37°C in an incubator with humidified atmosphere with 5% CO_2_. For cultivation under hypoxic condition, hypoxia chamber, and oxygen controller (BioSpherix, Parish, NY, USA) were used with 5% CO_2_, 1.5% O_2_, 37°C, and humidified atmosphere. N_2_ gas was injected to remove oxygen. For treatment in cultivation, 20 μM YC-1 (A.G. Scientific Inc., San Diego, CA, USA) as a HIF-1α inhibitor, 10^−8^ M agonists, and 10^−7^ M antagonists of ADM and ADM2 (Phoenix Pharmaceuticals, Burlingame, CA, USA) were used.

### Total RNA extraction and mRNA analysis

Total RNAs were extracted from placental tissues of NPE or PE and HTR-8/SVneo cells using TRIzol reagent (Ambion, Boston, MA, USA) following the manufacturer’s protocol. After RNA concentration was measured with a Nanodrop 2000 (Thermo Fisher Scientific, Waltham, MA, USA), cDNA was synthesized using Superscript III (Invitrogen, Carlsbad, CA, USA) and RNase-out (Invitrogen, Carlsbad, CA, USA) according to the manufacturer’s protocols. Then mRNA levels of target genes were determined by quantitative real-time polymerase chain reaction (qRT-PCR) using SYBR green master mix (Hoffmann-La Roche, Basel, Switzerland) and reverse transcription-PCR (RT-PCR) using h-Taq DNA Polymerase kit (SolGent, Daejeon, Korea) according to manufacturers’ protocols. Primer sequences used in this study are shown in Supplementary Table 1. Relative expression level of mRNA in qRT-PCR was analyzed with the comparative ΔΔCT method after normalization against level of β-actin as an internal control. For RT-PCR analysis, mRNA expression was measured by electrophoresis using 1.5% agarose gel and 6× loading dye. The intensity of photographed band was measured and calculated using ImageJ v1.43 (http://imagej.nih.gov/ij/;NIH, Bethesda, MD, USA). All experiments were performed in duplicates or triplicates.

### Total protein extraction and western blot analysis

Total proteins were extracted from placental tissues of NPE or PE and trophoblast cells. Homogenized tissues or cultivated cells were lysed using RIPA buffer (Sigma–Aldrich, St. Louis, MO, USA) supplemented with phosphatase inhibitor cocktail (A.G. Scientific Inc., San Diego, CA, USA) and protease inhibitor (Hoffmann-La Roche, Basel, Switzerland). Protein concentrations in lysates of tissues or cells were determined using BCA assay (Thermo Fisher Scientific, Waltham, MA, USA) using a microplate reader (Molecular Devices, San Jose, CA, USA). All tissue lysates were used after pooling in the same mass. For western blot, protein extracts were denatured and subjected to 10–15% Sodium Dodecyl Sulfate (SDS) polyacrylamide gel electrophoresis. SDS gels were transferred onto polyvinylidene difluoride (PVDF) membranes (Bio-Rad Laboratories, Inc., Berkeley, CA, USA). These transferred PVDF membranes were blocked with 5% bovine serum albumin (BSA; AMRESCO, Solon, OH, USA) or 5% skim milk (BD Biosciences, Heidelberg, Germany) for 1 h at room temperature. After overnight incubation with primary antibodies at 4°C, PVDF membranes were washed with phosphate-buffered saline including Tween-20 (PBS-T; elbio, Seoul, Korea). These membranes were then incubated with horseradish peroxidase-conjugated secondary antibodies at room temperature for 2 h. After washing PVDF membranes with PBS-T, they were developed with enhanced chemiluminescence (ECL) method using Clarity Western ECL substrate (Bio-Rad Laboratories, Inc., Berkeley, CA, USA). Bands intensity was analyzed using ImageJ v1.43 (NIH). Antibodies used in this study are shown in Supplementary Table 2. All experiments were performed in triplicate.

### Enzyme-linked immunosorbent assay

Enzyme-linked immunosorbent assay (ELISA) was performed to analyze levels of soluble MMP-9, sHLA-G, and HIF-1α. All ELISA experiments (soluble MMP9: R&D Systems, Minneapolis, MN, USA; sHLA-G: BioVendor, Brno-Řečkovice a Mokrá Hora, Czech; HIF-1α: Active Motif, Carlsbad, CA, USA) were proceeded according to manufacturers’ protocols. Briefly, cell supernatants, pregnant women-derived sera samples, and nuclear extracts prepared using nuclear fractionation (Active Motif, Carlsbad, CA, USA) were incubated in 96-well plates coated with binding targets: primary antibodies or specific binding nucleotide sequence. After washing, samples were reacted with secondary antibodies and washed again. Protein mass was determined using secondary antibody-specific development reagents. Optical density of each well of the plate was measured using a microplate reader (Molecular Devices, San Jose, CA, USA). All samples were loaded in duplicate or triplicate.

### Invasion assay

Trophoblast invasion ability was determined using a 24-well cell culture transwell system with pore size of 8 μm (Falcon, BD biosciences, Franklin Lakes, NJ, USA). After HTR-8/SVneo cells (3 × 10^4^) were added to the upper chamber with 0.3 mL serum-free medium whereas culture medium containing 5% FBS was added to the lower chamber, they were incubated for 24 h. To measure the number of invaded cells, insert membranes were fixed using 4× paraformaldehyde (PFA; elbio, Seoul, Korea) and 100% methanol (Ducksan, Seoul, Korea) and stained with Mayer’s Hematoxylin (Dako, Carpinteria, CA, USA). After washing with phosphate-buffered saline (PBS), stained membrane of transwell was mounted onto a slide glass (Marienfeld, Lauda-Königshofen, Germany) using mounting solution (Dako, Carpinteria, CA, USA) and fixed with a cover glass (Marienfeld, Lauda-Königshofen, Germany). Numbers of stained cells in at least nine randomly selected nonoverlapping fields were counted under a light microscope. All experiments were performed in triplicate.

### Statistical analysis

All experiments were performed in duplicate or triplicate. Some results were standardized using fold-change. Statistical significance was considered when *P*-value was less than 0.05 in Student *t-*test. All data and graphs are presented as mean ± standard error of mean (SEM).

## Results

### Expression levels of oxidative stress-related genes, ADM, and ADM2 in normal and preeclamptic placental tissues

**Figure 1:**
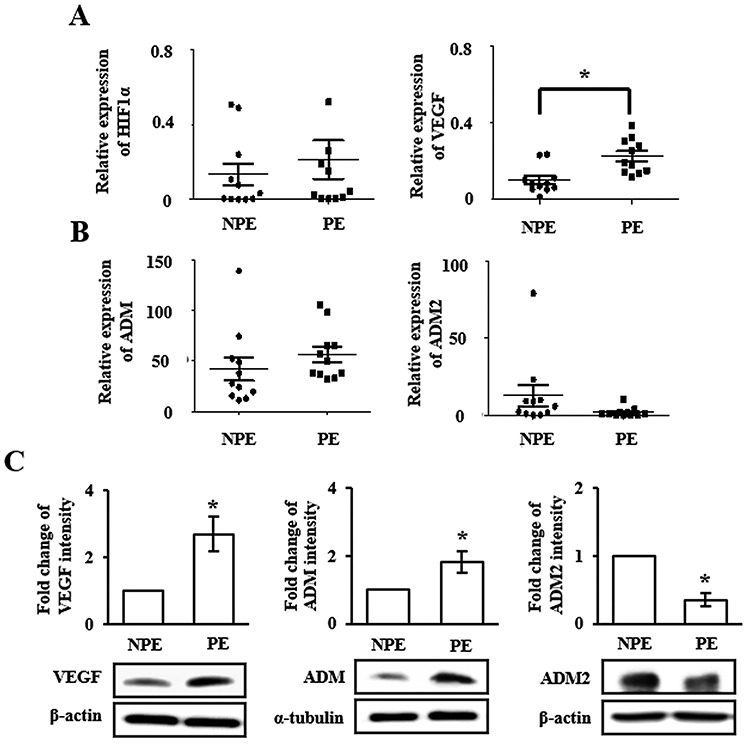
Expression of oxidative stress genes and ADM/ADM2 in preeclamptic (PE) placenta. (A) mRNA expression of HIF-1α and VEGF, (B) ADM and ADM2 using qRT-PCR, and (C) protein expression of VEGF (left), ADM (middle), and ADM2 (right) using western blot were examined in normal (NPE; N = 15) and 3rd trimester-PE (PE; N = 15) placenta. ^*^P < 0.05 of NPE vs. PE.

Hypoxia is a well-known risk factor for PE [6], increasing HIF-1α expression. It promotes expression of downstream genes such as VEGF related to tension and expansion of vasculature [10, 25]. In addition, dysfunction in endothelia caused by oxidative stress tends to induce PE [13]. To analyze characteristics of normal (NPE, n = 10) and 3rd trimester PE placental tissues (PE, n = 15), expression levels of oxidative stress-related genes HIF-1α and VEGF were determined using qRT-PCR and western blot analysis. Expression levels of HIF-1α and VEGF were significantly (*P* < 0.05) higher in PE compared to those in NPE (Figure 1A). These results indicate that upregulated mRNA levels of HIF-1α and VEGF are predominant conditions in PE induced by hypoxia. Also, both mRNA and protein expression levels of ADM were significantly increased whereas those of ADM2 were significantly decreased in PE compared to those in NPE (*P* < 0.05, Figure 1B and C). Although mRNA expression levels of invasion-related genes such as RhoA, Rac1, MMP2, and MMP9 varied in PE, protein expression levels of RhoA, Rac1, and MMP2 were significantly increased in PE compared to those in NPE (*P* < 0.05, Supplementary Figure 1A–I). These results suggest that increased expression of HIF-1α and VEGF by oxidative stress can mediate expression levels of ADM and ADM2 as well as migration genes differently in placental tissues.

### Dual role of hypoxia on expression of ADM and ADM2 in human trophoblast cell line, HTR-8/SVneo cells

**Figure 2:**
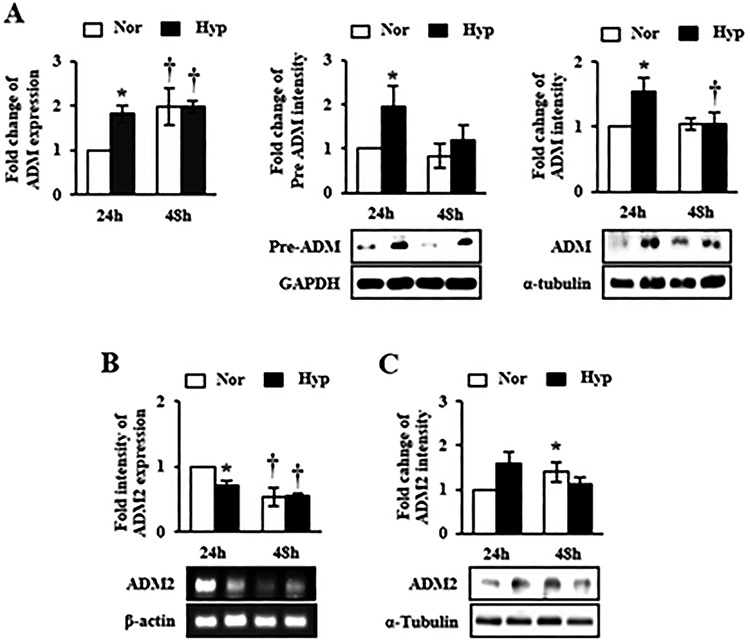
Expression of ADM and ADM2 in HTR-8/SVneo, a trophoblast cell line, under hypoxia. HTR-8/SVneo cells were cultured under normoxic and hypoxic conditions for 24 h and 48 h. (A) mRNA levels of ADM (left) and protein levels of ADM-precursor (Pre-ADM, middle) and ADM (right) were increased after 24 h of incubation of cells under hypoxia. (B) ADM2 mRNA expression was decreased whereas (C) its protein level was not changed after exposure to hypoxia for 24 h. ^*^P < 0.05 of normoxia vs. hypoxia; †*P* < 0.05 of 24 h vs. 48 h of incubation.

To investigate whether expression levels of ADM and ADM2 were affected by hypoxia, trophoblast cells HTR-8/Svneo were cultured under normoxic or hypoxic conditions for 24 and 48 h. After exposure to hypoxic condition for 24 h, expression levels of HIF-1α in HTR-8/SVneo cells were significantly increased (*P* < 0.05, Supplementary Figure 2A). Expression levels of mRNA and protein of both ADM-precursor (pre-ADM) and ADM were also increased when cells were cultured under hypoxic conditions for 24 h (*P* < 0.05, Figure 2A) compared to those in cells cultured under normoxic conditions. Migration-related genes were also upregulated under hypoxic conditions (Supplementary Figure 2B–E). On the other hand, mRNA level of ADM2 was decreased (Figure 2B) whereas its protein expression level seemed to be increased under hypoxic condition for 24 h (Figure 2C). These results suggest that hypoxia plays a dual role in modulating ADM and ADM2 expression in HTR-8/SVneo cells.

**Figure 3:**
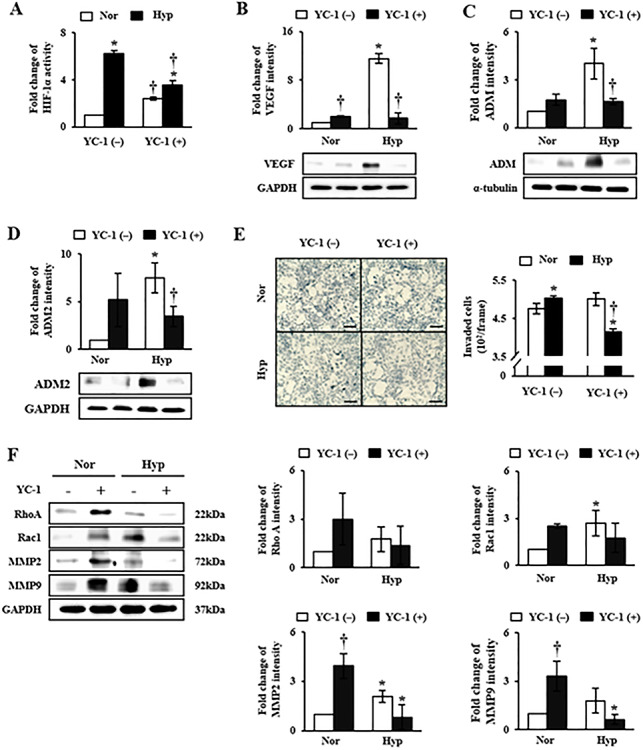
Alteration of ADMs expression and migration of HTR-8/SVneo cells caused by HIF-1α inhibition using YC-1. In the analysis for (A) HIF-1α activity using ELISA, and (B) protein expression of VEGF using western blot in HTR-8/SVneo cells, YC-1 (20 μM) treatment inhibited HIF-1α activity under hypoxia. Likewise, protein expression levels of (C) ADM and (D) ADM2 were down-regulated by YC-1 treatment under hypoxia according to western blot assay. (E) Increased invasion ability of HTR-8/SVneo cells in hypoxia was diminished by YC-1 treatment in invasion assay. (F) Protein expression levels of invasion-related genes such as RhoA (middle-upper), Rac1 (right-upper), MMP2 (middle-lower), and MMP9 (right-lower) were altered by treatment with YC-1 under hypoxic conditions. Scale bar indicates 100 μm in length. ^*^P < 0.05 of normoxia vs. hypoxia; †*P* < 0.05 of YC-1 (−) vs. YC-1 (+).

### Expression levels of ADM and ADM2, and migration activity of trophoblast cells are regulated via HIF-1α dependent manner in hypoxic condition

In previous reports, we demonstrated that hypoxia can increase the migration activity of trophoblast cells by increasing migration-related factors [26]. Also, we confirmed that expression levels of HIF-1α, ADM, and ADM2 in HTR-8/SVneo cells were increased under hypoxic condition in the study. So, we hypothesized whether the altered expressions of ADM and ADM2 by HIF-1α could be controlled migration activity of trophoblast cells. To determine whether HIF-1α could alter expression levels of ADM and ADM2 or migration activity of HTR-8/SVneo cells, levels of ADM and ADM2 and migration activities of HTR-8/SVneo cells under hypoxia were examined after inhibiting HIF-1α by treatment with YC-1, a compound known to inhibit HIF-1α. As shown in Figure 3A, expression level of HIF-1α under hypoxia was significantly down-regulated by YC-1 compared to that in untreated control group (*P* < 0.05, Figure 3A). VEGF expression under hypoxia was also significantly decreased by YC-1 treatment (*P* < 0.05, Figure 3B). These results confirmed the YC-1 treatment effectively reduce HIF-1α activity in HTR-8/SVneo cells under hypoxia. At the same time, increased expression levels of ADM and ADM2 under hypoxia were down-regulated by YC-1 treatment (*P* < 0.05, Figure 3C and D). In addition, the invasion ability of HTR-8/SVneo cells under hypoxia was higher than that under normoxic condition. However, YC-1 treatment significantly decreased the invasion ability of HTR-8/SVneo cells under hypoxia (*P* < 0.05, Figure 3E). Although YC-1 treatment resulted in increased expression levels of migration-related proteins such as Rac1, MMP2, and MMP9 in HTR-8/SVneo cells under normoxic condition, their expression levels were significantly decreased under hypoxic condition after YC-1 treatment (*P* < 0.05, Figure 3F). These findings indicate that expression levels of ADM and ADM2 and migration ability of trophoblast cells are regulated via HIF-1α-dependent manner in hypoxic condition.

### Effects of ADM and ADM2 on invasion ability of trophoblast cells

**Figure 4:**
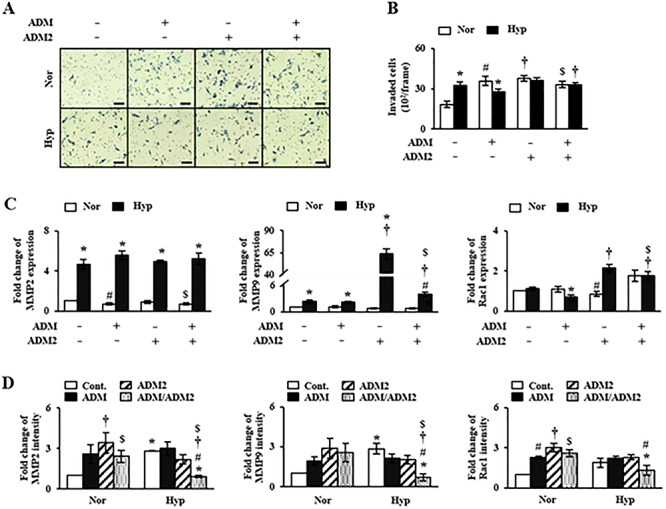
Invasion ability of HTR-8/SVneo cells after cotreatment with ADM and ADM2 agonists. According to results of invasion assay (A, B), invasion ability of HTR-8/SVneo cells was activated by treatment with ADM and ADM2 agonists under normoxic condition, but not under hypoxia. (C) mRNA and (D) protein expression levels of MMP2 (left), MMP9 (middle), and Rac1 (right) in HTR-8/SVneo cells after treatment with ADM and ADM2 agonists under hypoxic conditions were analyzed by qRT-PCR and western blot, respectively. Each expression at hypoxia with cotreatment of ADM and ADM2 agonists was decreased in common except for mRNA level of MMP2. However, these events were not shown under normoxia. Scale bar indicates 100 μm in length. ^*^P < 0.05 of normoxia vs. hypoxia; #*P* < 0.05 of ADM(−) vs. ADM(+); †*P* < 0.05 of ADM2(−) vs. ADM2(+); $*P* < 0.05 of control vs. cotreatment of ADM and ADM2.

To investigate effects of ADM and ADM2 on invasion ability of trophoblast cells, we analyzed invasion ability of HTR-8/SVneo cells after treatment with single or combined agonist of ADM and ADM2. As shown in Figure 4A and B, invasion ability of agonist-untreated cells under hypoxic condition was higher compared to that under normoxic condition. Invasion activity of HTR-8/SVneo cells after treatment with agonist under normoxia condition was also higher than that of the untreated group (*P* < 0.05). In contrast, invasion activity of HTR-8/SVneo cells after treatment with ADM agonist under hypoxia was lower compared to that under normoxic condition after treated with ADM agonist. Interestingly, after treatment with both ADM and ADM2 agonists under hypoxia, ADM2 treatment showed a tendency to rescue the decreased invasion activity of HTR-8/SVneo cells caused by ADM treatment under hypoxia condition compared to the untreated hypoxia group.

To determine whether ADM and AMD2 were involved in the control of invasion activity of trophoblast cells, expression levels of MMP2, MMP9, and Rac1 genes in HTR-8/SVneo cells were analyzed after treatment with AMD and AMD2 agonists. MMP2 mRNA expression level was not significantly altered by agonist treatment under normoxic or hypoxic condition (Figure 4C). However, mRNA expression levels of MMP9 and Rac1 under hypoxic condition were dramatically increased by ADM2 agonist treatment compared to those in untreated or ADM agonist treated group under hypoxic condition. In particular, expression level of MMP9 in ADM2 agonist treated group under hypoxic condition was increased more than 20-fold compared to that in the untreated group under hypoxic condition (*P* < 0.05, Figure 4C). Interestingly, the effect of ADM2 agonist on MMP9 and Rac1 expression was reduced by treatment with both agonists (*P* < 0.05, Figure 4C and D; Supplementary Figure 2E) under hypoxic condition. These results suggest that balanced interaction between ADM and ADM2 by hypoxia might play an important role in trophoblast invasion through increasing MMP9 expression as well as ADM2 agonist can increase the invasion of trophoblast whereas ADM agonist can attenuate the role of ADM2.

### Regulation of trophoblast migration by ADM and ADM2 depending on hypoxia

**Figure 5:**
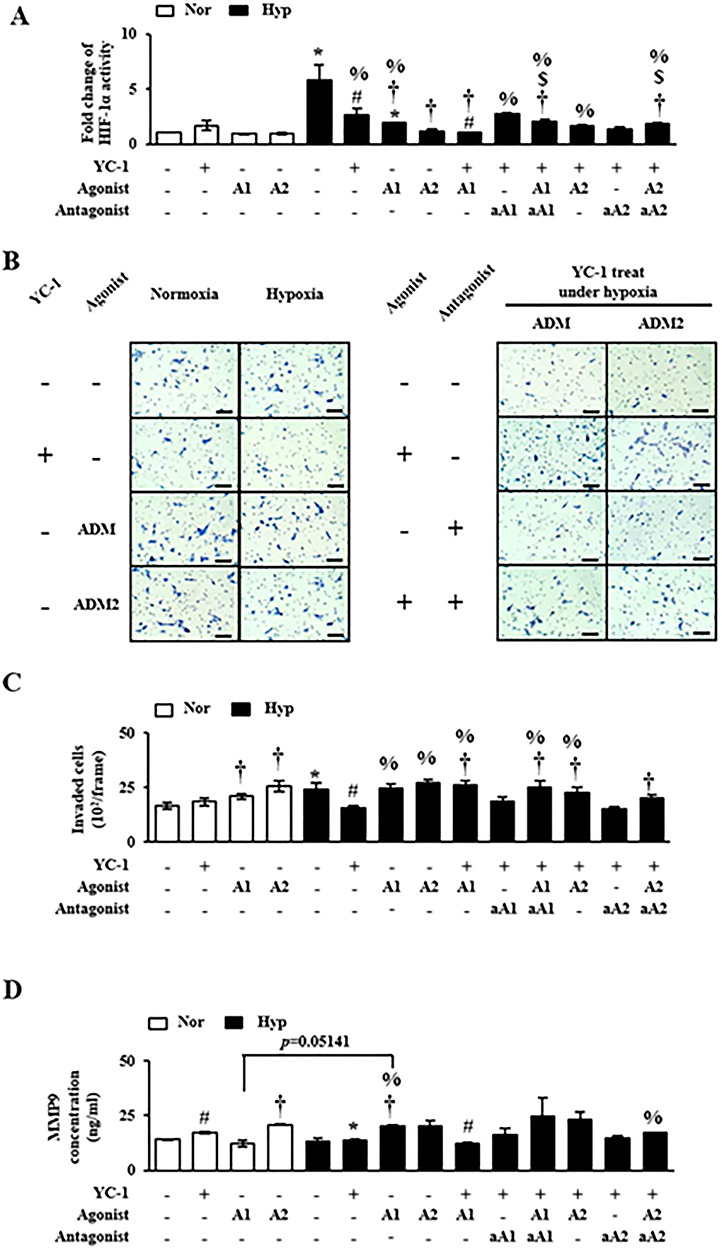
Regulation of trophoblast function by YC-1, ADM, and ADM2 agonists/antagonists in hypoxic condition. (A) Activity of HIF-1α in HTR-8/SVneo cells was measured by ELISA after treatment with YC-1 and agonists and/or antagonists of ADM and ADM2. HIF-1α activity induced by hypoxia was decreased by treatment with agonists of ADM and ADM2 along with YC-1. Even in the presence of YC-1 under hypoxia, HIF-1α activity was altered by agonists and antagonists of ADM and ADM2. (B, C) Invasion ability of HTR-8/SVneo cells was inhibited by YC-1 treatment, but rescued by treatment with ADM and ADM2 agonists. (D) MMP9 concentration in cell culture media was measured using MMP9 ELISA, suggesting that agonists for ADM and ADM2 could induce MMP9 expression. ^*^P < 0.05 of normoxia vs. hypoxia; #*P* < 0.05 of YC-1 (−) vs. YC-1 (+); †*P* < 0.05 of agonist (−) vs. agonist (+); $*P* < 0.05 of antagonist (−) vs. antagonist (+); %*P* < 0.05 of normoxia control vs. others.

Our results confirmed that the expression of ADM and ADM2 was controlled by increased HIF-1α under hypoxic condition. In addition, balanced interaction of ADM and ADM2 affected the invasion activity of trophoblast under hypoxia. To determine whether ADM and ADM2 could affect HIF-1α activity and the trophoblast migration depending on hypoxia, expression levels of HIF-1α were examined after treatment with various combinations of YC-1, agonist or antagonist of ADMs under normoxia or hypoxia. As shown in Figure 5A, there was no significant difference in HIF-1α activity among normoxic conditions with or without treatment by YC-1 and agonists. However, agonist treatment significantly decreased HIF-1α activity even more than YC-1 treatment under hypoxic condition (*P* < 0.05, Figure 5A) whereas down-regulated HIF-1α activities by YC-1 and agonist of ADMs were gradually rescued by addition of antagonist for ADM or ADM2 (*P* < 0.05, Figure 5A). In invasion assay of HTR-8/SVneo cells after treatment with YC-1, agonist and antagonist were also used to elucidate the role of ADM and ADM2 in trophoblast invasion under hypoxia. As expected, decreased invasion activity of trophoblast cells by YC-1 treatment was recovered by agonist treatment under hypoxic condition (*P* < 0.05, Figure 5B and C). These dynamic regulations of ADM and ADM2 after treatments with agonist and antagonist induced differences in invasion ability of trophoblast cells. The effects seemed to be linked to activities of MMP9 in their culture medium based on ELSIA analysis (Figure 5D). These results suggest that balanced interaction between ADM and ADM2 can affect HIF-1α activity and trophoblast migration depending on hypoxic condition.

**Figure 6:**
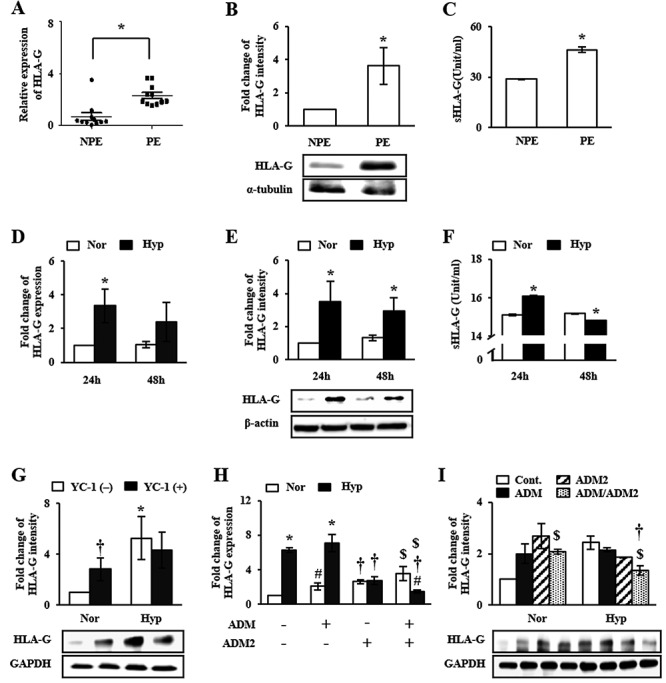
HLA-G expression in preeclamptic placental tissues and HTR-8/SVneo cells. (A, D) mRNA and (B, E) protein expression levels of HLA-G were analyzed using qRT-PCR and western blot, respectively. (C, F) sHLA-G protein was analyzed using ELISA. As shown in comparisons of normal (NPE) and 3rd trimester-PE (PE), mRNA expression of HLA-G was altered by hypoxia, but its protein levels were increased. (G) HLA-G expression in HTR-8/SVneo cells treated with YC-1 under hypoxia was attenuated. In results of (H) mRNA and (I) protein levels of HLA-G in HTR-8/SVneocells after combined treatment with ADM and ADM2 agonists, combinatorial treatment increased HLA-G expression under normoxia, but not under hypoxia. Also, under normoxia, mRNA expression of HLA-G was elevated by singular treatment of each agonist, but not under hypoxia. Especially, ADM2 agonist showed negative effect on HLA-G expression under hypoxia. ^*^P < 0.05 of NPE vs. PE or normoxia vs. hypoxia; †*P* < 0.05 of 24 h vs. 48 h, YC-1(−) vs. YC-1(+), or ADM2(−) vs. ADM2(+); #*P* < 0.05 of ADM(−) vs. ADM(+); $*P* < 0.05 of control vs. ADM/ADM2.

### Effects of ADM and ADM2 on HLA-G expression in trophoblast cells

HLA-G is an immune tolerance factor that plays a role in fetal and maternal interaction during pregnancy [20]. Trophoblast cells express HLA-G, a key factor for successful invasion by minimizing immune rejection while invading into the endometrial layer of maternal uterus. Next, we examined whether ADM and AMD2 could affect the expression of HLA-G and how the correlation of ADM, ADM2, and HLA-G might regulate invasion ability of trophoblast cells under hypoxic condition. Although mRNA expression level of HLA-G was decreased in PE (Figure 6A), protein expression level of HLA-G in placental tissue and concentration of soluble HLA-G (sHLA-G) in serum of PE women were significantly increased, compared to those in NPE (*P* < 0.05, Figure 6B and C). In addition, both mRNA and protein expression levels of HLA-G (Figure 6D and E), and sHLA-G in culture medium (Figure 6F) of HTR-8/SVneo cells were increased (*P* < 0.05) when these cells were exposed to hypoxic culture condition for 24 h. Increased HLA-G expression in trophoblast cells might be associated with elevated HIF-1α activity by oxidative stress. These results suggest that oxidative stress can contribute to the enhancement of HLA-G expression by affecting ADM and ADM2. As shown in Figure 6G, the increase of HLA-G expression in HTR-8/SVneo cells induced by the hypoxic condition was attenuated by YC-1 treatment, suggesting an important role of HIF-1α in HLA-G expression. Furthermore, we evaluated HLA-G mRNA expression in HTR-8/SVneo cells after treatment with ADM and ADM2 agonists. In hypoxic condition, the increase of HLA-G expression in trophoblast cells was maintained regardless of ADM treatment whereas mRNA expression level of HLA-G in ADM2-treated group was decreased (*P* < 0.05, Figure 6H). Especially, HLA-G expression was markedly decreased in the group cotreated with ADM and ADM2 agonists under hypoxia compared to that in the untreated hypoxia group (*P* < 0.05, Figure 6H and I). These results suggest that HIF-1α expression induced by hypoxia can trigger alternative expression of ADM and ADM2 as a positive and negative regulator, respectively, resulting in increased invasion activity of trophoblast cells and HLA-G expression.

## Discussion

Trophoblast cells play a major role in implantation and formation of the maternal–fetal interface during pregnancy. In early pregnancy, physiological low oxygen tension is important for the differentiation of trophoblast cells and regulation of cellular events such as invasion ability and altered expression of various genes [26, 27]. Failure in function of trophoblasts is associated with several diseases such as PE and IUGR. It is well known that insufficient trophoblast invasion into maternal spiral arteries in the endometrium of maternal uterus contributes to the development of PE which is also associated with hypoxia and oxidative stress in the placenta [9, 10, 27]. It has been reported that trophoblast cells cultured under hypoxic conditions show improvement of various functions according to changes in gene expression [6]. Increased HLA-G expression and invasion ability of trophoblast cells cultured under hypoxic conditions have been demonstrated in our previous study and other studies [28, 29].

ADM and ADM2 belong to a unique group of CGRP family. They are potent vasodilators with structural similarity and overlapping biological effects, although they have different potencies [30]. Recently, Chauhan et al. have reported that ADM2 can regulate invasion activity of trophoblast cells and HLA-G expression [17, 20]. However, no studies have reported that expression levels of ADM and ADM2 are regulated by hypoxia in HIF-1α-dependent manner and that balanced interaction of these peptides can influence the invasion ability of trophoblast cells under hypoxic condition. Due to the reason, we demonstrated that expression levels of ADM and ADM2 were differently regulated in PE placental tissues: ADM expression was increased whereas ADM2 expression was decreased (Figure 1). In addition, expression of ADM was increased in trophoblast cells cultured under hypoxic condition. However, expression levels of ADM2 and invasion-related genes in trophoblast cells line (i.e., HTR-8/SVneo) cultured under hypoxic condition were partially different from those in PE placental tissues (Figures 1 and 2). Therefore, it is necessary to identify the cause of inhibition of ADM2 expression in PE patients. Our studies suggested that ADM was also involved in invasion activity of trophoblast cells line (i.e., HTR-8/SVneo) and HLA-G expression, showing function similar to ADM2. Like ADM, a well-known hypoxia-related gene, ADM2 expression in trophoblast cells was also regulated by hypoxia. Therefore, hypoxia plays a dual role in modulation of ADM and ADM2. Based on these data, we confirmed that inhibition of HIF-1α signaling by YC-1 treatment prevented increases of expression levels of these peptides and invasion ability of trophoblast cells under hypoxia (Figure 3). This result shows that expression of ADM and ADM2 in trophoblast cells is regulated via HIF-1α-dependent manner in hypoxia. In addition, the invasion ability of trophoblast cells under hypoxia was influenced by combined treatment with ADM and ADM2 agonists, resulting in downregulation of invasion-related factors MMP9 and Rac1 that could lead to functional deterioration of trophoblast cells. Furthermore, increased HIF-1α by hypoxia affected balanced interaction of ADM and ADM2 as well as trophoblast migration (Figure 4).

Generally, ADM and ADM2 share a signal through different combinations of the CRLR and one of three RAMPs (RAMP1, RAMP2, or RAMP3) and that effects of ADM are mediated by CRLR and RAMP2 or RAMP3 whereas effects of ADM2 are mediated by CRLR and RAMP3 [14, 18, 30]. These overlapping or different CRLR-RAMP interactions with ADM and ADM2 may cause conformational variation and consequently affect specific gene expression and functional changes of trophoblast cells. We also confirmed similar effects resulting from the recovery of the invasion ability which was reduced after treatment with antagonists of ADM or ADM2. Such effect was rescued by correspondent agonist.

Expressing HLA-G in trophoblast plays a role in immune tolerance to facilitate the interaction with maternal immune cells and endothelial cells. It also plays a role in the regulation of trophoblast migration. In addition, hypoxia can change HLA-G expression in trophoblast cells and tumor cells whereas there is no reduction of HLA-G expression in cytotrophoblasts by short-term exposure to hypoxia [29, 31, 32]. In the present study, we confirmed that mRNA expression level of HLA-G in trophoblast cells was increased by hypoxia in HIF-1α dependent manner, but decreased in trophoblast cells after cotreatment with ADM and ADM2 agonists. Increased HLA-G protein expression and secretion in PE were similar to those in trophoblast under hypoxic condition. These results suggest that expression of HLA-G in PE might be due to change of HIF-1α expression and alterative expression of ADM and ADM2.

In summary, our results indicate that HIF-1α expression induced by hypoxia can trigger altered expression of ADM and ADM2, resulting in increased invasion activity of trophoblast cells and HLA-G expression. These results also suggest that the activity of ADM and ADM2 might lead to various changes in the physiology of trophoblast cells such as limitation of HIF-1α activity, invasion of trophoblast cells, and HLA-G expression. These effects are dependent on the balance of ADMs. Therefore, ADM and ADM2 could be used as an indicator for diagnosis of obstetric diseases including PE. They might also help us understand the fundamental mechanism of the pathophysiology of PE.

## Supplementary Material

Supple_Fig_1-Gu_et_al_ioaa178

Revised_supplementary_figure_2_Gu_et_al_ioaa178

Revised_supplementary_figure_3_Gu_et_al_ioaa178

revised_Supplementary_Table_Gu_et_al_ioaa178
