# Supplementary figures and images for: Altered expression of ADM and ADM2 by hypoxia regulates migration of trophoblast and HLA-G expression
Source: Biol Reprod. 2020 Sep 30;104(1):159–69. doi: 10.1093/biolre/ioaa178 (PMC7786263; doi:10.1093/biolre/ioaa178)

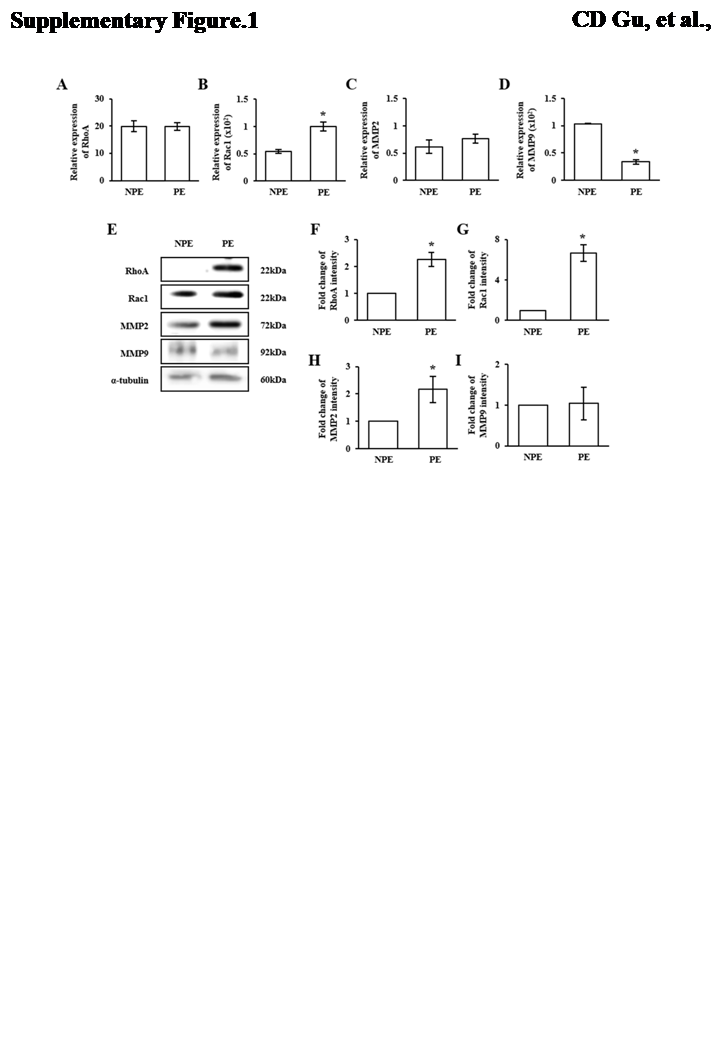

Supplement: Supple_Fig_1-Gu_et_al_ioaa178 [file supple_fig_1-gu_et_al_ioaa178.png]

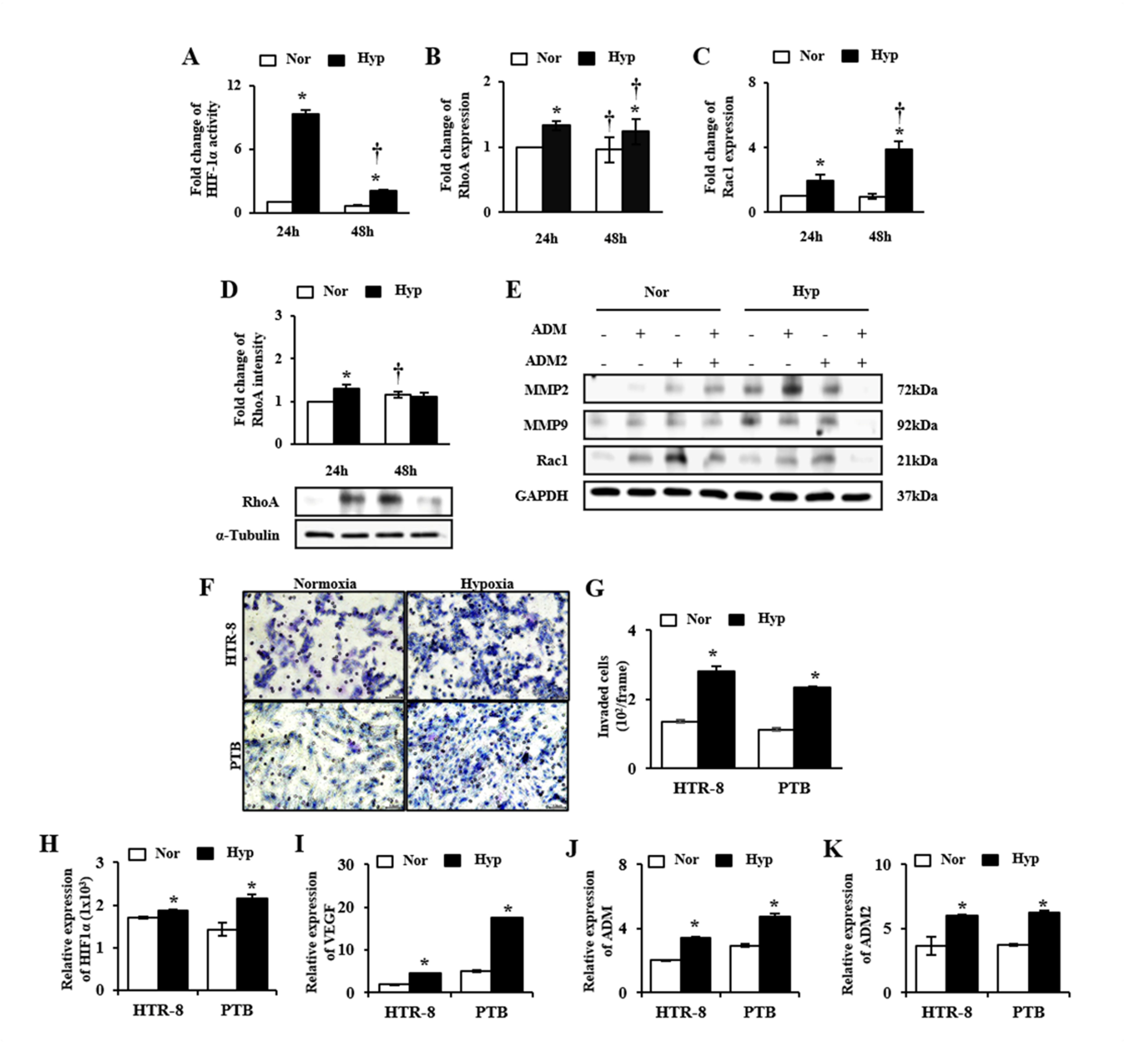

Supplement: Revised_supplementary_figure_2_Gu_et_al_ioaa178 [file revised_supplementary_figure_2_gu_et_al_ioaa178.png]

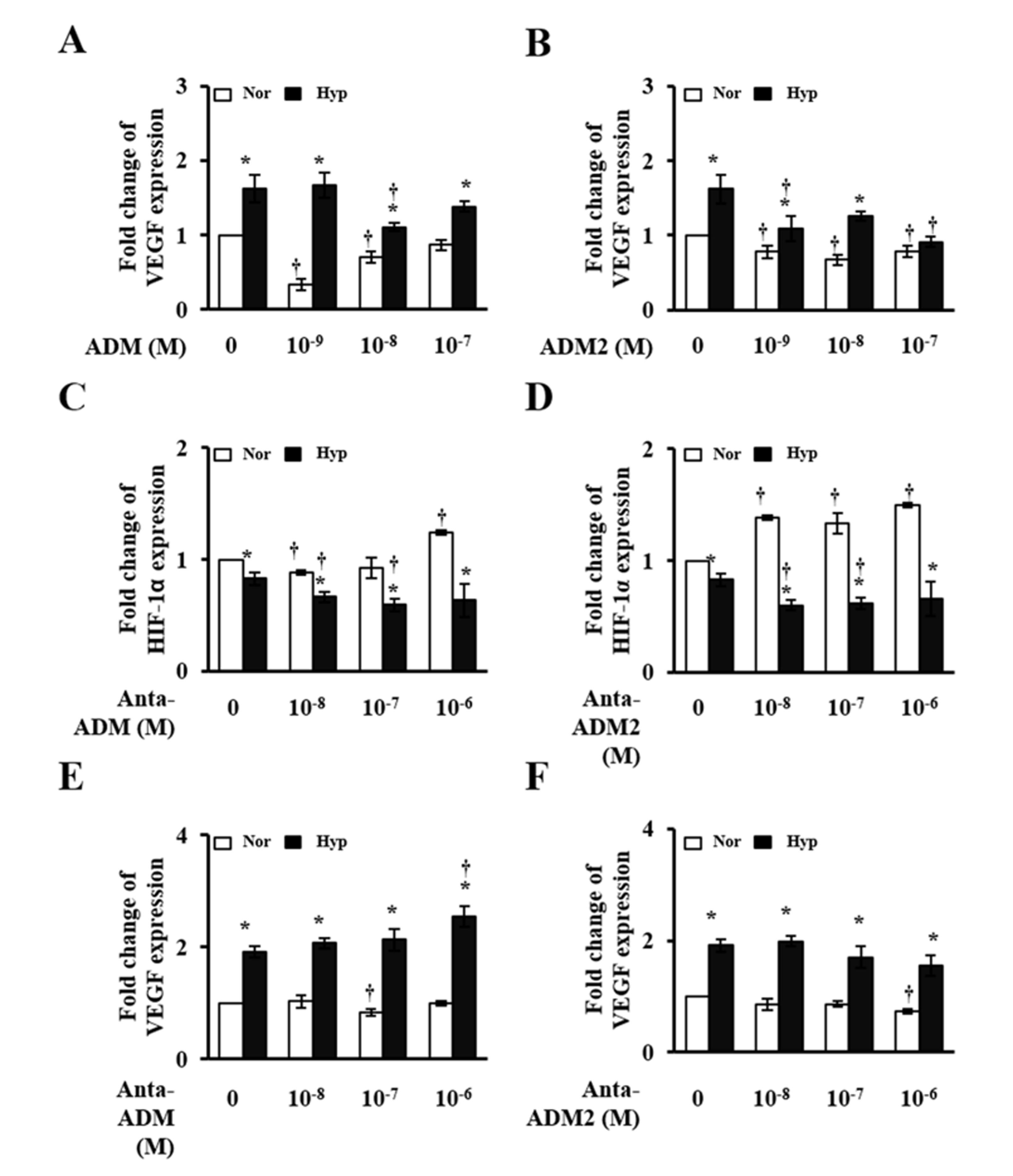

Supplement: Revised_supplementary_figure_3_Gu_et_al_ioaa178 [file revised_supplementary_figure_3_gu_et_al_ioaa178.png]
