# Supplementary material for: Altered expression of ADM and ADM2 by hypoxia regulates migration of trophoblast and HLA-G expression
Source: Biol Reprod. 2020 Sep 30;104(1):159–69. doi: 10.1093/biolre/ioaa178 (PMC7786263; doi:10.1093/biolre/ioaa178)
Supplement: revised_Supplementary_Table_Gu_et_al_ioaa178 [file revised_supplementary_table_gu_et_al_ioaa178.doc]

**Supplementary table 1.**Primers used in this study.

| **Gene** | **Sequence** | **Reference(PMID)**  **Or**  **Gene accession** | **Size (bp)** | **Tm (℃)** |
| --- | --- | --- | --- | --- |
| **ADM** | F: 5'-GGAAGAGGGAACTGCGGATGT-3' | 25439669 | 137 | 60 |
|  | R: 5'-GGCATCCGGACTGCTGTCT-3' |  |  |  |
| **ADM2** | F: 5'-TGTTATGGGTCAGCCTCTCG-3' | 17008878 | 138 | 54 |
|  | R: 5'-TGGCTGAGATTCTGCACCTGG-3' |  |  |  |
| **HIF-1α** | F: 5'-GTTTACTAAAGGACAAGTCA-3' | 16912095 | 193 | 60 |
|  | R: 5'-TTCTGTTTGTTGAAGGGAG-3' |  |  |  |
| **RAC1** | F: 5'-TGATGCAGGCCATCAAGTGT-3' | NM 006908.4 | 237 |  |
|  | R: 5'-AGAACACATCTGTTTGCGGATAG-3' |  |  |  |
| **HLA-G** | F: 5'-CTGACCCTGACCGAGACCTG-3' | 21787741 | 329 |  |
|  | R: 5'-GTCGCAGCCATCATCCACTGGAG-3' |  |  |  |
| **β-actin** | F: 5'-GGGACCTGACTGACTACCTCAT-3' | 15841445 | 109 |  |
|  | R: 5'-ACGTAGCACAGCTTCTCCTTAAT-3' |  |  |  |
| **RhoA** | F: 5'-TGGAAAGCAGGTAGAGTTGG-3' | NM 001664.3 | 163 |  |
|  | R: 5'-GACTTCTGGGGTCCACTTTT-3' |  |  |  |
| **VEGF** | F: 5'-GCCTTGCCTTGCTGCTCTAC-3' | 21325301 | 106 |  |
|  | R: 5'-ACATCCATGAACTTCACCACTTCG-3' |  |  |  |
| **MMP2** | F: 5'-CGGCCGCAGTGACGGAAA-3' | 18612129 | 212 |  |
|  | R: 5'-CATCCTGGGACAGACGGAAG-3' |  |  |  |
| **MMP9** | F: 5'-GACGCAGACATCGTCATCCAGTTT-3' | 18612129 | 200 |  |
|  | R: 5'-GCCGCGCCATCTGCGTTT-3' |  |  |  |

**Supplementary table 2.** Antibodies used in this study.

| Primary Antibody | | | | | | | | | |
| --- | --- | --- | --- | --- | --- | --- | --- | --- | --- |
| Target protein | Cat.# | Company | | Predicted size (kDa) | | Host | Application | | Titer (dilution) |
| ADM | sc-33787 | Santa Cruz | | 6 active,  22 precursor | | Rabbit | Hu, Mu, Rt | | 500 |
| ADM2 | sc-86272 | Santa Cruz | | 16 | | Rabbit | Hu | | 500 |
| HLA-G | NB110-55297 | Novus | | 39 | | Mouse | Hu | | 1000 |
| Rac1 | NBP1-61994 | Novus | | 21 | | Rabbit | Hu, Mu, Rt | | 1000 |
| RhoA | ab68826 | Abcam | | 22 | | Rabbit | Hu, Mu, Rt, Dg | | 1000 |
| VEGF | MAB293-110 | R&D | | 22, 42 | | Mouse | Hu | | 1000 |
| MMP2 | IM33 | Calbiochem | | 72 latent,  66 active | | Mouse | Hu, Mu, Rt | | 1000 |
| MMP9 | IM09L | Calbiochem | | 92 latent,  83 active | | Mouse | Hu | | 1000 |
| MMP9 | IM37 | Calbiochem | | 92 latent,  83 active | | Mouse | Hu, Rt, Rb | | 1000 |
| β-actin | A1978 | Sigma | | 42 | | Mouse | Hu | | 3000 |
| GAPDH | LF-PA00212 | Abfrontier | | 37 | | Rabbit | Hu | | 3000 |
| α-tubulin | CP06-100UG | oncogene | | 60 | | Mouse | Hu | | 3000 |
| Secondary Antibody | | | | | | | | | |
| Target protein | | | Cat.# | | Company | host | application | Titer(dilution) | |
| Goat anti-mouse IgG HRP conjugated | | | 170-5047 | | Bio-Rad | Goat | Mouse | 10000, 25000 | |
| Goat anti-rabbit IgG HRP conjugated | | | 170-5046 | | Rio-Rad | Goat | Rabbit | 10000, 25000 | |
